# The Role of Transcatheter Arterial Embolization in Traumatic Pelvic Hemorrhage: Not Only Pelvic Fracture

**DOI:** 10.7759/cureus.722

**Published:** 2016-08-03

**Authors:** Alessio Comai, Marianna Zatelli, Thomas Haglmuller, Giampietro Bonatti

**Affiliations:** 1 Department of Radiology, Hospital of Bolzano; 2 Department of Anesthesiology, Hospital of Bolzano

**Keywords:** hemorrhage, trauma, embolization, interventional radiology, pelvic fractures

## Abstract

Purpose: The most common life-threatening complication of pelvic trauma is bleeding. Arterial bleedings frequently require active management, preferably with transcatheter arterial embolization (TAE). Hemodynamic instability and/or contrast extravasation at computer tomography (CT) examination are reliable indicators of arterial injury. Unstable pelvic fractures are much more hemorrhagic than stable fractures. Nevertheless, an absent or isolated pelvic fracture does not exclude pelvic hemorrhage.

Materials and Methods: A retrospective study was conducted on our institutional database by collecting data of patients who underwent pelvic angiography and/or embolization due to pelvic blunt trauma in the period between August 2010 and August 2015.

Results: In a period of five years, 39 patients with traumatic pelvic bleeding underwent angiography at our institution. Thirty-six of the 39 (92%) patients did show CT signs of active pelvic bleeding. Nineteen of 39 (49%) patients were hemodynamically unstable at presentation. Three of the 39 patients did not require embolization. Technical success was 35/36 (97%), and overall mortality was 3/39 (8%). Notably, 5/39 (13%) patients did not have any pelvic fracture at presentation, and 18/39 (46%) had only isolated or stable pelvic ring fracture.

Conclusions: TAE is an effective technique to treat arterial pelvic bleeding after trauma. The absence of a major pelvic fracture does not exclude the risk of active bleeding requiring prompt treatment.

## Introduction

The most common life-threatening complication of acute pelvic trauma is bleeding. Management of patients with pelvic fractures is challenging and is principally based on hemodynamic status and associated injuries. Hemorrhage can occur from cancellous bony surfaces exposed by fractures, presacral venous plexus lacerations, and iliac arterial or venous branches [1]. Moreover, pelvic fractures are often associated with hemorrhages from other locations, such as chest, abdomen, or long bones. The mortality rate among patients with pelvic fractures is 16% [2]. Arterial hemorrhage can also occur without pelvic fracture, as sporadically reported. Arterial bleedings frequently require active management, preferably with transcatheter arterial embolization (TAE) [3-5]. TAE is an effective technique for facilitating rapid hemostasis at active arterial bleeding sites with a reported success rate ranging from 85% to 100%. Hemodynamic instability and/or contrast extravasation at computer tomography (CT) examination are reliable indicators of arterial injury [6]. In patients with pelvic fractures, contrast extravasation on the arterial phase CT scans is encountered frequently; thereafter, the need for intervention seems to be determined by the presence of clinical signs of ongoing bleeding [7]. To improve survival of hemodynamically unstable patients with pelvic fractures, the hemorrhage must be stopped immediately [8]. An alternative treatment for hemodynamic unstable pelvic fractures is pelvic packing, which mainly controls venous bleeding and bleeding from the fracture sites [9]. There is still no clear consensus about the best strategy to adopt with patients presenting with active pelvic bleeding [10]. This paper aims to evaluate the efficacy of TAE in the management of pelvic bleeding and to correlate the presence and type of pelvic fracture with clinical severity and outcome.

## Materials and methods

A retrospective study was conducted using our institutional database by collecting data from all the patients who underwent pelvic angiography and/or embolization due to pelvic blunt trauma in the period between August 2010 and August 2015. Approval of this retrospective study was granted by the Institutional Review Board of our institute, the District of Bolzano/Bozen, Alto Adige/Suedtirol Healthcare Company. Informed consent for angiography and eventual embolization was obtained from patients whenever possible. Data, including age, gender, presence, the type of pelvic fracture according to the Young and Burgess classification [11], hemodynamic parameters before and after endovascular treatment (arterial pressure, heart rate), and serum hemoglobin levels before and after treatment. Pelvic fractures were classified into three groups: 1) absent pelvic fracture; 2) isolated bone or stable pelvic ring fracture; and 3) unstable pelvic ring fracture, including anteroposterior (AP) type II and III, lateral compression (LC) type III, and vertical (V) and complex (C) types. Statistical analysis was conducted to correlate patient characteristics with admission vitals, the presence and severity of the pelvic fracture, and clinical outcome.

All patients underwent total body CT scan, except one, according to our institutional protocol for polytrauma patients on a Dual Source CT system or a 16-slice CT system (SOMATOM Definition Flash and Sensation 16, Siemens, Erlangen, Germany). This protocol consists of a precontrast head scan, arterial phase from skull base to proximal thighs, portal venous phase on the abdomen, and an optional late venous phase/excretory phase.

Indications for angiography were CT signs of active bleeding or arterial injuries and hemodynamic instability, defined as systolic blood pressure inferior to 90 mm Hg. In hemodynamically stable patients with signs of ongoing bleeding at CT, the decision to perform embolization was made by a group consensus of the interventional radiologist, surgeon, and intensivologist team. In the presence of unstable pelvic ring fractures, the first approach was the pelvic ring stabilization with a T-POD® pelvic binder (Pyng Medical, Richmond, Canada) or a C-clamp external fixation.

Digital subtraction angiography (DSA) examinations were performed with a Multistar system (Siemens AG, Erlangen, Germany) before 2013 and with an Allura Clarity system (Philips, Eindhoven, Netherlands) after 2013. All angiographic procedures were performed by one member of a team composed of four interventional radiologists available on a 24-hour basis. The preferred access site was the common right femoral artery. After a lower abdominal aorta angiogram, selective angiograms of pelvic arteries were obtained according to angiographic findings or previously evaluated CT images. Contrast extravasation, pseudoaneurysms, arteriovenous fistulas, and cut-off vessel signs were all considered as arterial injuries requiring embolization. Treatment was considered technically successful when all arterial injuries were excluded at post-embolization angiograms. Clinical success was defined as the percentage of patients who were hemodynamically stable after treatment.

## Results

In a five year time period (August 2010-August 2015), 39 patients with traumatic pelvic bleeding underwent angiography. The group was composed of 24 males and 15 females with an average age of 53 years (range: 17-94). The median age was significantly different in relation to sex (p < 0.001): median age was 47 in the male group and 81 in the female group. All patients had CT signs of pelvic hematoma: three patients (8%) did not show active contrast material extravasation, whilst 36/39 (92%) did show CT signs of active pelvic bleeding. Nineteen of 39 patients (49%) were hemodynamically unstable at presentation, as defined by a systolic blood pressure inferior to 90 mmHg; 20/39 patients (51%) were hemodynamically stable. Table 1 reports the characteristics, admission vitals, and outcome of hemodynamically stable vs unstable patients.

**Table 1:** Patient Data Considering Hemodynamic Stability at Presentation Clinical failure is defined as hemodynamic instability after angiography and, when applied, embolization. n = number; SBP = systolic blood pressure; Hb = serum hemoglobin (g/dL)

	Stable	Unstable	All
	n = 20	n = 19	n = 39
		median (range) or n (%)	
Age (years)	48 (17-83)	59 (22-94)	53 (17-94)
Female	5 (25%)	10 (53%)	15 (38%)
Admission vitals			
SBP	117 (90-155)	69 (40-85)	94 (40-155)
Heart rate	83 (60-100)	99 (70-130)	91 (60-130)
Hb	10 (6.4-16.9)	7.2 (2-14.1)	8.7 (2-16.9)
Post-treatment			
Clinical failure, n (%)	0 (0%)	4 (21%)	4 (10%)
Mortality, n (%)	0 (0%)	3 (16%)	3 (8%)

Three of 39 patients were not treated with embolization because of the absence of contrast extravasation at selective angiograms and hemodynamic stability. All the other patients underwent embolization of one or more target vessels. Technical success was 35/36 (97%), as one patient succumbed during intervention due to cardiac arrest.

Five of 39 patients (13%) without any pelvic fracture underwent angiography because of the presence of a pelvic hematoma with active extravasation at CT; three of them were hemodynamically unstable at presentation and became stable after embolization. There was no correlation between the presence, type of fracture, and hemodynamic parameters at presentation, as reported in Table 2.

**Table 2:** Patient Data Considering Pelvic Fracture Type Fractures are divided into three groups: no fracture; isolated pelvic fractures (pubic ramus, transverse sacrum) and stable pelvic fractures (anteroposterior I, lateral compression I-II); and unstable pelvic fractures (anteroposterior II-III, lateral compression III, vertical, and complex). Clinical failure is defined as hemodynamic instability after angiography and, if applied, embolization. n = number; PRF = pelvic ring fracture; Hb = serum hemoglobin (g/dL); SBP = systolic blood pressure

	None	Isolated or stable PRF	Unstable PRF	All
	n = 5	n = 18	n = 16	n = 39
		median (range) or n (%)		
Age (years)	66 (45-94)	61 (17-92)	41 (18-84)	53 (17-94)
Female	2 (40%)	8 (44%)	5 (31%)	15 (38%)
Admission vitals				
SBP	90 (60-115)	102 (60-155)	85 (40-120)	94 (40-155)
Hemodynamic instability	3 (60%)	8 (44%)	8 (50%)	19 (49%)
Heart rate	96 (80-110)	87 (60-130)	93 (70-120)	91 (60-130)
Hb	8.7 (6-10)	10 (7.7-15)	9.5 (5-11.2)	8.7 (2-16.9)
Post-treatment				
Clinical failure, n (%)	1 (20%)	0 (0%)	3 (19%)	4 (10%)
Mortality, n (%)	0 (0%)	0 (0%)	3 (19%)	3 (8%)

Mortality rate was 3/39 (8%): all the deceased patients belonged to the hemodynamically unstable group at presentation and to the unstable pelvic ring fracture (PRF) group (in both groups, p = 0.1; Fisher's exact test). Hemodynamic stability was obtained or preserved in 35/39 patients (90%); one patient required reintervention due to bleeding from another pelvic source. Clinical success in the hemodynamic instability group was 15/19 (79%). Clinical failure was strongly associated with hemodynamical instability at presentation (p < 0.01; McNemar test).

## Discussion

The pelvic ring is much more solid than many other bone structures, and high-energy trauma is required to disrupt this complex. Consequently, these fractures are rarely found in isolation, and patients with pelvic fractures often have multiple trauma [12]. The seriousness of pelvic fractures lies in the possible occurrence of retroperitoneal hematoma and hemorrhagic shock [13]. High mortality rates in patients with blunt pelvic trauma are due to pelvic hemorrhage in the first 24 hours following trauma; conversely, later deaths can be associated with multiple organ failure (MOF). This phenomenon is directly linked to blood transfusion as well as increased ICU length of stay and mortality rate caused by the bloody vicious cycle [14].

Approximately 3% of patients with pelvic fracture present with an arterial injury requiring embolization [15]. The type of fracture can influence the clinical outcome since patients with open pelvic fractures have an increased mortality rate up to 55%. In particular, arterial injuries are predominantly observed in an unstable pelvic fracture as anterior-posterior pelvic compression type II and III, type III lateral compression injuries, vertical compression injuries, and combined injury mechanisms. Unstable pelvic fractures are much more hemorrhagic than stable fractures [16]. Moreover, they have been reported to be a predictor of the need for hemostatic embolization [17-18]. Most available papers describe cohorts of patients with complex or unstable pelvic fractures and pelvic hemorrhage who undergo angiography and eventual embolization. Nevertheless, an absent or isolated pelvic fracture does not exclude pelvic hemorrhage as it has been sporadically reported [19-22]. In our experience, five patients without pelvic fracture underwent angiography because of the CT presence of a pelvic hematoma with active extravasation. Notably, three of them were hemodynamically unstable at presentation and became stable after embolization. The remaining two patients were hemodynamically stable at presentation: in one case, angiography was negative and treatment was not performed; in the other, a blush was confirmed and treated.

Although the data are not statistically significant, all deceased patients presented with pelvic ring instability (p = 0.1; Fisher's exact test). Brun, et al. have recently reported that unstable fractures, according to the Burgess and Young classification, were not associated with an increased requirement for pelvic angiography [23]. Similarly, other research groups did not find any correlation between pelvic ring integrity and embolization or hemodynamic shock [24-25]. Although this argument is still controversial, it is important to note that most of the reported study groups consider pelvic fracture as a selection criterion. On the other side, cases of pelvic bleeding without pelvic fracture are sporadically reported.

Hemodynamic instability at presentation showed an association with clinical failure defined as the percentage of hemodynamically unstable patients despite treatment. This phenomenon could probably be due to treatment delay and the consequent bloody vicious cycle [14]. Hemodynamic status on arrival is reported to be a major criterion that dictates the immediate management of the patient [4]. Management of hemodynamically stable patients is controversial, as it can lead to an overtreatment. As the presence and severity of a pelvic fracture have limited predictive value for the patient's need of endovascular treatment, an accurate contrast-enhanced dynamic CT study should always be conducted to demonstrate active bleeding, its entity, and localization [22].

Current Advanced Trauma Life Support® (ATLS) advice that CT is contraindicated in hemodynamically unstable patients has been questioned [26]. ATLS recommends FAST scanning (focused assessment sonography in trauma) or diagnostic peritoneal lavage and pelvic radiogram for such patients [27]. Nevertheless, in a retrospective multicenter cohort study on 16,719 major trauma patients, whole-body CT increased survival in hemodynamically stable as well as hemodynamically unstable patients [28]. CT imaging provides additional characterization of injury as contrast extravasation, presence, and localization of pelvic hematoma. Although these CT findings did not result as reliable predictors of positive angiography, their absence did not reliably exclude pelvic bleeding and the need for angiography [17]. Moreover, Charbit, et al. reported that one-third of hypotensive patients with a large hemoperitoneum did not show any active intraperitoneal hemorrhage [29]. However, multiple detector computed tomography (MDCT) findings correlate strongly with DSA findings, though sensitivity remains imperfect [30]. This modality is particularly helpful to identify the likely source of hemorrhage, allowing the interventional radiologist to perform a faster and more selective embolization. In clinical practice, MDCT is particularly important in the absence of angiographic signs of bleeding when clinical signs indicate intermittently active bleeding.

The presented study examines a relatively low number of cases, which hampers the possibility to significantly correlate fractures with hemodynamic data at presentation and with clinical outcome. Moreover, it is limited by the retrospective design with the inherent suboptimal data collection and selection bias.

## Conclusions

TAE is an effective technique to treat traumatic pelvic bleeding in the presence of clinical and/or CT signs of active or intermittent bleeding. We suggest that the absence of a major pelvic fracture does not exclude the risk of active bleeding, requiring prompt treatment. Conversely, hemodynamic instability at presentation is associated with mortality despite treatment.
